# Functional Recovery in Autoimmune Encephalitis: A Prospective Observational Study

**DOI:** 10.3389/fimmu.2021.641106

**Published:** 2021-05-21

**Authors:** Thomas Seifert-Held, Katharina Eberhard, Christian Lechner, Stefan Macher, Harald Hegen, Tobias Moser, Gregor Brecl Jacob, Gertraud Puttinger, Raffi Topakian, Michael Guger, Emrah Kacar, Lea Zoche, Desiree De Simoni, Andreas Seiser, Stefan Oberndorfer, Christoph Baumgartner, Walter Struhal, Friedrich Zimprich, Johann Sellner, Florian Deisenhammer, Christian Enzinger, Markus Reindl, Helmut Rauschka, Thomas Berger, Romana Höftberger

**Affiliations:** ^1^ Department of Neurology, Medical University of Graz, Graz, Austria; ^2^ Core Facility Computational Bioanalytics, Center for Medical Research, Medical University of Graz, Graz, Austria; ^3^ Division of Pediatric Neurology, Department of Pediatrics I, Medical University of Innsbruck, Innsbruck, Austria; ^4^ Division of Neuropathology and Neurochemistry, Department of Neurology, Medical University of Vienna, Vienna, Austria; ^5^ Department of Neurology, Medical University of Vienna, Vienna, Austria; ^6^ Department of Neurology, Medical University of Innsbruck, Innsbruck, Austria; ^7^ Department of Neurology, Christian Doppler Medical Center, Paracelsus Medical University, Salzburg, Austria; ^8^ Department of Neurology, University Medical Centre Ljubljana, Ljubljana, Slovenia; ^9^ Department of Neurology 1, Kepler University Hospital, Johannes Kepler University, Linz, Austria; ^10^ Department of Neurology, Academic Teaching Hospital Wels-Grieskirchen, Wels, Austria; ^11^ Department of Neurology 2, Kepler University Hospital, Johannes Kepler University, Linz, Austria; ^12^ Department of Neurology, University Clinic Tulln, Karl Landsteiner University of Health Sciences, Tulln, Austria; ^13^ Department of Neurology, Hospital Hietzing, Vienna, Austria; ^14^ Department of Neurology, University Clinic St. Pölten, Karl Landsteiner University of Health Sciences, St. Pölten, Austria; ^15^ Karl Landsteiner Institute for Clinical Epilepsy Research and Cognitive Neurology, Vienna, Austria; ^16^ Medical Faculty, Sigmund Freud University, Vienna, Austria; ^17^ Department of Neurology, Landesklinikum Mistelbach-Gänserndorf, Mistelbach, Austria; ^18^ Department of Neurology, Hospital Donaustadt, Vienna, Austria; ^19^ Karl Landsteiner Institute for Neuroimmunological and Neurodegenerative Diseases, Vienna, Austria

**Keywords:** autoimmune encephalitis, anti-NMDAR-encephalitis, anti-LGI1-encephalitis, anti-CASPR2-encephalitis, modified Rankin Scale

## Abstract

**Background:**

Prospective observations of functional recovery are lacking in patients with autoimmune encephalitis defined by antibodies against synaptic proteins and neuronal cell surface receptors.

**Methods:**

Adult patients with a diagnosis of autoimmune encephalitis were included into a prospective registry. At 3, 6 and 12 months of follow-up, the patients’ modified Rankin Scale (mRS) was obtained.

**Results:**

Patients were stratified into three groups according to their antibody (Ab) status: anti-NMDAR-Ab (n=12; group I), anti-LGI1/CASPR2-Ab (n=35; group II), and other antibodies (n=24; group III). A comparably higher proportion of patients in group I received plasma exchange/immunoadsorption and second line immunosuppressive treatments at baseline. A higher proportion of patients in group II presented with seizures. Group III mainly included patients with anti-GABA_B_R-, anti-GAD65- and anti-GlyR-Ab. At baseline, one third of them had cancer. Patients in groups I and III had much higher median mRS scores at 3 months compared to patients in group II. A median mRS of 1 was found at all follow-up time points in group II.

**Conclusions:**

The different dynamics in the recovery of patients with certain autoimmune encephalitides have important implications for clinical trials. The high proportion of patients with significant disability at 3 months after diagnosis in groups I and III points to the need for improving treatment options. More distinct scores rather than the mRS are necessary to differentiate potential neurological improvements in patients with anti-LGI1-/CASPR2-encephalitis.

## Introduction

Autoimmune encephalitides defined by antibodies against synaptic proteins and neuronal cell surface receptors have emerged as specific diagnostic entities (1–3). Clinical presentation includes focal neurologic, neuropsychiatric and/or cognitive symptoms and/or impaired consciousness. Immunosuppressive treatments improve the patients’ clinical outcome based on uncontrolled observations (4–6). A small randomized trial has shown superiority of intravenous immunoglobulins (IVIG) to placebo in reducing seizure frequency in anti-leucine-rich, glioma-inactivated 1-antibody (anti-LGI1-Ab) and anti-contactin-associated protein-like 2-antibody (anti-CASPR2-Ab) seropositive adult patients (7). Other randomized trials evaluating the efficacy of IVIG, rituximab and bortezomib are ongoing (8–10). We have set up a multicenter prospective observational study for a structured assessment of diagnostics and treatments applied in clinical routine in adult patients with autoimmune encephalitis. Focus is on the patients’ functional outcome assessed by the modified Rankin Scale (mRS) (11) at pre-specified follow-up intervals. Previous observational studies were mainly retrospective and have applied a wide range of observational periods with heterogenous outcome measures (12).

## Patients and Methods

### Study Cohort

Eleven clinical centers in Austria and one in Slovenia, covering a population of about 9 million people, included patients in a prospective registry with the following inclusion and exclusion criteria:

Inclusion criteria:

- Patients ≥18 years of age.
- mRS score ≤1 before the appearance of first clinical symptoms related to autoimmune encaephalitis, i.e. patients without pre-existing disability.
- mRS score ≥2 at maximum of the disease
- Occurrence of first clinical symptoms related to autoimmune encephalitis ≤ 6 months before inclusion into the study.
- A diagnosis of autoimmune encephalitis based on clinical presentation according to established criteria and either anti-glutamic acid decarboxylase 65-antibodies (anti-GAD65-Ab) or specific neuronal surface antibodies in serum and/or cerebrospinal fluid (CSF) (2): anti-N-methyl-D-aspartate receptor-antibodies (anti-NMDAR-Ab), anti-LGI1-Ab, anti-CASPR2-Ab, anti-alpha-amino-3hydroxy-5-methyl4-isoxazolepropionic acid receptor-antibodies (anti-AMPAR-Ab), anti-gamma-aminobutyric acid A and B receptor-antibodies (anti-GABA_A_R-Ab, anti-GABA_B_R-Ab), anti-metabotropic glutamate receptor 1 and 5-antibodies (anti-mGluR1-Ab, anti-mGluR5-Ab), anti-glycin-receptor-atibodies (anti-GlyR-Ab), anti-dipeptidyl-peptidase-like protein-6-antibodies (anti-DPPX-Ab), anti-IgLON5-antibodies (anti-IgLON5-Ab), anti-Dopamine 2 receptor-antibodies, anti-Neurexin 3-alpha-antibodies (1). Methods and scope of antibody testing in individual patients were applied on discretion of the treating neurologists. Although GAD65 is an intracellular protein, patients with anti-GAD65-Ab share certain characteristics with surface autoimmunity and were therefore included in this study.
- According to established criteria, a diagnosis of autoimmune encephalitis in patients negative for antibodies in serum and CSF mentioned above (2).

Exclusion criteria:

- Patients with infectious encephalitis.
- Steroid-responsive encephalopathy with autoimmune thyreoiditis (SREAT).
- Detection of onconeural antibodies in peripheral blood.

### Data Acquisition and Statistical Analysis

Data entry into the prospective registry started at 01/01/2016 and is ongoing. Data extraction for this analysis was done on 29/10/2020. The patients’ demographics, clinical symptoms, results from antibody testing, CSF analysis, brain imaging, intensive care unit (ICU) admissions, tumor diagnostics, immunosuppressive, antiepileptic and anti-tumor treatments were recorded at baseline and on follow-up examinations at 3, 6 and 12 months. On all these follow-up examinations, the modified Rankin Scale (mRS) was obtained as used in the majority of published outcome studies in autoimmune encephalitis (12). Although there is known interobserver variability (13), the patients’ mRS was rated by neurologists experienced in the routine use of this scale in acute stroke care. Immunosuppressive treatments were ranked as first line (high-dose intravenous corticosteroids, oral corticosteroids, intravenous immunoglobulins, plasma exchange, immunoadsorption) or second line (rituximab, azathioprine, mycophenolate mofetil, cyclophosphamide i.v. or p.o., bortezomib) as established previously (4). Source data were entered into a web-based electronic data capture system which conforms to the Code of Federal Regulations Title 21 Part 11.

One-way analysis of variance (ANOVA) and the Chi-square test were applied for multi-group comparisons with post-hoc two group comparisons by the Games-Howell test. A p-value less than 0.05 was considered as statistically significant. In Chi-square tests, the deviation of the observed from the expected frequency was assessed by calculating adjusted standardized residuals where magnitudes above |1.96| correspond to p-values less than 0.05 and magnitudes above |2.58| correspond to p-values less than 0.01 (14). Friedman’s test with post-hoc two-group comparisons by Wilcoxon’s signed rank test and Bonferroni correction was applied for longitudinal changes in median mRS scores. Herein, a p-value less than 0.0167 was considered statistically significant. The Chi-square test was used for sample size calculations with a two-sided significance level of 0.05 and a power of 80%. Sample size calculations were performed using nQuery (Statsols, Cork, Ireland). All other statistical analyses were performed using IBM SPSS statistics version 26 (IBM, Chicago, Illinois, USA).

## Results

**Figure 1:**
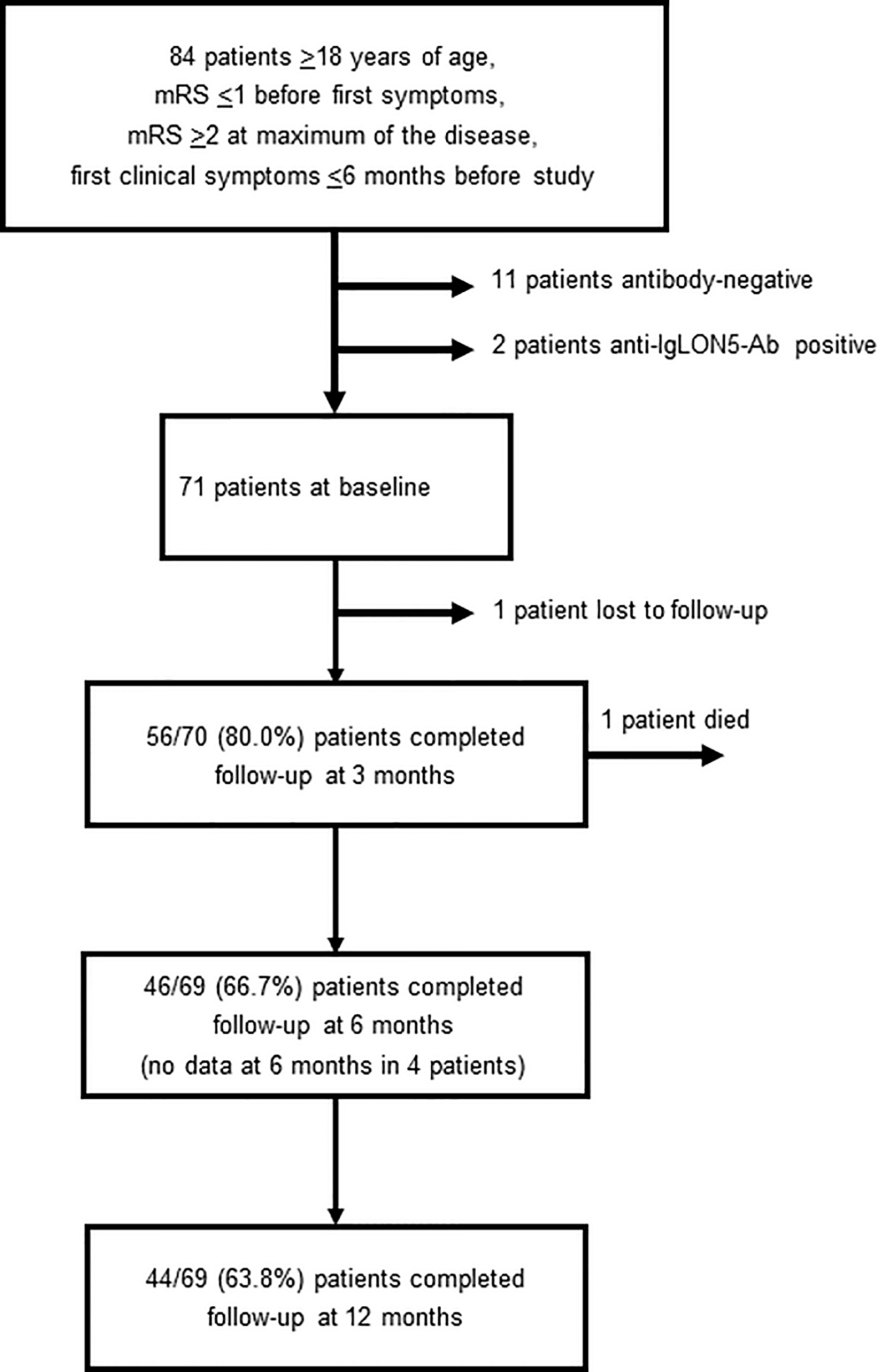
Study flow chart (Ab, antibodies; mRS, modified Rankin scale).

A total of 84 patients fulfilled the inclusion criteria. 11 patients negative for antibodies against synaptic proteins and neuronal cell surface receptors were excluded from the analysis. Another 2 patients with anti-IgLON5-Ab were excluded. Anti-IgLON5 disease follows a chronic progressive course, and response to immunotherapy is variable (15). 71 patients were included into the analysis: 41 men, 30 women, mean age 53.5 years (range 18-81 years), 69 (97.2%) Caucasians. On the day of data extraction for this analysis, 56, 46 and 44 patients, respectively, have completed follow-up examinations at 3, 6 and 12 months (**Figure 1**). One patient was lost to follow-up after baseline. One patient died until 3 months of follow-up. Patients with antibodies against neuronal cell surface proteins and the synaptic protein GAD65 in serum and/or CSF were identified at baseline as follows: 12 (16.9%) anti-NMDAR-Ab, 24 (33.8%) anti-LGI1-Ab, 10 (14.1%) anti-CASPR2-Ab, 1 (1.4%) anti-LGI1-Ab and anti-CASPR2-Ab, 1 (1.4%) anti-AMPAR-Ab, 1 (1.4%) anti-GABA_A_R-Ab, 5 (7.0%) anti-GABA_B_R-Ab, 9 (12.7%) anti-GAD65-Ab, 1 (1.4%) anti-GABA_B_R-Ab and anti-GAD65-Ab, 7 (9.9%) anti-GlyR-Ab. For further analysis, patients were stratified according to their antibody status: one group of patients with anti-NMDAR-Ab (n=12), one with anti-LGI1/CASPR2-Ab (n=35), and one with all other antibodies (n=24). The study flow chart (**Figure 1**) shows the total number of patients who have completed 3, 6 and 12 moths of follow-up at the day of data extraction for this analysis. One patient with anti-LGI1-Ab was lost to follow-up after baseline. For another patient with LGI1-Ab, data are not available at 6 but at 12 months follow-up. For 3 patients in the group with other antibodies, data are not available at 6 but at 12 months.

Patient demographics, clinical symptoms and tumor status at baseline are given in **Table 1**. Patients with anti-NMDAR-Ab were significantly younger, and psychosis was more often found as compared to other groups. Two patients with anti-NMDAR-Ab had a previous aseptic meningoencephalitis 8 and 10 years before, both at the age of 17 years. In one of them, anti-NMDAR-Ab were found retrospectively in a stored CSF specimen thereby identifying the previous disease as anti-NMDAR-encephalitis. Another patient in this group aged 40 years had suffered from herpes simplex encephalitis at the age of 3. No other previous encephalitides were reported in the patients in this study. In the group of patients with anti-NMDAR-Ab, a teratoma was found in 3 women at first presentation and in one woman within 12 months of follow-up, i.e. a teratoma was identified in 50% of all women in this group. Teratomas were all excised shortly after diagnosis.

**Table 1:** Demographic, clinical and paraclinical data of patients at baseline stratified according to their antibody status.

	anti-NMDAR-Abn = 12	anti-LGI1/CASPR2-Abn = 35	other Abn = 24	p
**Mean (SD) age, years**	31.9 (10.5)^a^	62.1 (12.0)^b^	51.7 (16.4)^b^	<0.001
**Women, n (%)**	8 (66.7)	9 (25.7)**	13 (54.2)	0.016
**Prodromal symptoms, n (%)**	7 (58.3)**	4 (11.4)**	7 (29.2)	0.005
**Psychosis, n (%)**	6 (50.0%)*	6 (17.1)	4 (16.7)	0.044
**Anxiety, n (%)**	4 (33.3)	10 (28.6)	0**	0.011
**Sleep disturbance, n (%)**	2 (16.7)	5 (14.3)	1 (4.2)	0.391
**Altered behaviour, n (%)**	3 (25.0)	4 (11.4)	3 (12.5)	0.488
**Seizures, n (%)**	4 (33.3)	23 (65.7)**	5 (20.8)**	0.002
**Status epilepticus, n (%)**	3 (25.0)	1 (2.9)	3 (12.5)	0.074
**Reduced consciousness, n (%)**	0	3 (8.6)	2 (8.3)	0.578
**Speech disorder, n (%)**	2 (16.7)	1 (2.9)	0	0.055
**Memory impairment, n (%)**	5 (41.7)	18 (51.4)	12 (50.0)	0.840
**Movement disorder, n (%)**	2 (16.7)	4 (11.4)	6 (25.0)	0.393
**Limb weakness, n (%)**	2 (16.7)*	0	1 (4.2)	0.047
**Autonomic dysfunction, n (%)**	4 (33.3)	5 (14.3)	2 (8.3)	0.143
**Respiratory insufficiency, n (%)**	3 (25.0)	2 (5.7)	4 (16.7)	0.172
**ICU admission, n (%)**	5 (41.7)	5 (14.3)	6 (25.0)	0.138
**MRI T2 mesial** **temporal hyperintensity, n (%)**	1 (8.3)	15 (42.9)*	5 (20.8)	0.040
**CSF WBC >20/µl, n (%)**	6 (50)**	1 (2.9)**	4 (16.7)	0.001
**Diagnosis of tumor at** **baseline, n (%)**	3 (25.0)	0*	8 (33.3)*	0.001

^a^p < 0.001 for all post-hoc two-group comparisons.

^b^p = 0.029 for post-hoc two-group comparison.

*adjusted standardised residual >|1.96|, equivalent to p-value < 0.05.

**adjusted standardised residual >|2.58|, equivalent to p-value < 0.01. Ab, antibodies; ICU, intensive care unit; MRI, magnetic resonance imaging; CSF WBC, cerebrospinal fluid white blood cell counts.

In the group of patients with anti-LGI1/CASPR2-Ab, two thirds of them presented with seizures. Other characteristics included a higher patients’ age, magnetic resonance imaging T2 mesial temporal hyperintensity in two fifths of patients, and a high proportion of CSF samples with low or normal cell counts (5). No tumors were found in these patients. In contrast, in the group of patients with other antibodies, one third had a tumor at first presentation of autoimmune encephalitis. Five of these patients suffered from lung cancer (4 with anti-GABA_B_R-Ab and one with anti-GAD65-Ab), one patient with anti-GABA_B_R-Ab had colon cancer, one patient with anti-GABA_A_R-Ab had thymoma, and one with anti-GlyR-Ab had breast cancer. One further patient with anti-GAD65-Ab was diagnosed with B-cell lymphoma at 3 months follow-up. One of the patients with anti-GABA_B_R-Ab and lung cancer died within 3 months of diagnosis. At least one follow-up examination was available in another 7 of these patients. Five of them received chemotherapy for lung cancer, breast cancer or lymphoma. In the patients with colon cancer, thymoma and breast cancer, surgery was performed. The patient with breast cancer also received radiotherapy.

Immunosuppressive and antiepileptic treatments applied at baseline and at 3, 6 and 12 months of follow-up are shown in **Table 2**. Almost all patients received first line immunosuppression at baseline. A significantly higher proportion of patients with anti-NMDAR-Ab received plasma exchange or immunoadsorption at baseline. Second line immunosuppressive treatments were applied at baseline in about four fifths of patients with anti-NMDAR-Ab and in one fifth of patients with anti-LGI1/CASPR2-Ab and other antibodies.

**Table 2:** Immunosuppressive and antiepileptic treatments applied in patients stratified according to their antibody status.

	anti-NMDAR-Ab	anti-LGI1/CASPR2-Ab	other Ab	p
**1^st^ line IST at baseline including PE/IA, n (%)**	12/12 (100)	34/35 (97.1)	21/24 (87.5)	0.187
**PE/IA at** **baseline, n (%)**	7/12 (58.3)**	3/35 (8.6)**	7/24 (29.2)	0.002
**2^nd^ line IST at baseline, n (%)**	10/12 (83.3)**	8/35 (22.9)	5/24 (20.8)	<0.001
**AET at** **baseline, n (%)**	9/12 (75.0)	26/35 (74.3)	10/24 (41.7)**	0.025
**1^st^ line IST at** **3 months, n (%)**	6/11 (54.5)	22/28 (78.6)	11/17 (64.7)	0.296
**2^nd^ line IST at** **3 months, n (%)**	5/11 (45.5)*	2/28 (7.1)*	4/17 (23.5)	0.023
**AET at** **3 months, n (%)**	5/11 (45.5)	17/28 (60.7)	6/17 (35.3)	0.241
**1^st^ line IST at** **6 months, n (%)**	4/10 (40.0)	15/24 (62.5)	7/12 (58.3)	0.478
**2^nd^ line IST at** **6 months, n (%)**	5/10 (50.0)*	2/24 (8.3)*	3/12 (25.0)	0.026
**AET at** **6 months, n (%)**	5/10 (50.0)	15/24 (62.5)	4/12 (33.3)	0.253
**1^st^ line IST at** **12 months, n (%)**	3/10 (30.0)	11/23 (47.8)	5/11 (45.5)	0.627
**2^nd^ line IST at** **12 months, n (%)**	4/10 (40.0)	6/23 (26.1)	4/11 (36.4)	0.683
**AET at** **12 months, n (%)**	4/10 (40.0)	15/23 (65.2)*	2/11 (18.2)*	0.032

*adjusted standardised residual >|1.96|, equivalent to p-value < 0.05.

**adjusted standardised residual >|2.58|, equivalent to p-value < 0.01. Ab, antibodies; IST, immunosuppressive treatments; AET, antiepileptic treatments; PE, plasma exchange; IA, immunoadsorption.

The patients’ functional outcome at 3, 6 and 12 months of follow-up is given in **Table 3**. Functional outcome at 3 months was significantly better in the group of patients with anti-LGI1/CASPR2-Ab as reflected by a lower median mRS and a higher proportion of patients who have recovered to mRS <2. Two thirds of patients with anti-LGI1/CASPR2-Ab recovered to mRS scores <2 at 3 months, whereas only about 30% of patients with anti-NMDAR-Ab or other Ab have recovered at 3 months. These proportions increased to 50% at 6 months for both groups (**Table 3**). Based on these proportions of patients who have recovered at the different time points during follow-up, sample size calculations for treatment trials were performed. Any potential new treatment that increases the proportion of patients who recover to mRS <2 at 3 months from 30% (standard of care group) to 50% (new treatment group) would require a sample size in each group of 93 patients (Odds ratio 2.333; 0.05 two-tailed significance; 80% power). Accordingly, studies for treatments which increase the proportion of patients who recover to mRS <2 at 6 months from 50% to 70% would require the same sample sizes.

**Table 3:** Functional outcome at 3, 6 and 12 months of follow-up in patients at the time of data extraction for this study.

	anti-NMDAR-Ab	anti-LGI1/CASPR2-Ab	other Ab	p
**mRS <2 at** **3 months, n (%)**	3/11 (27.3)	19/28 (67.9)**	6/17 (35.3)	0.026
**mRS (median) at** **3 months**	3.0	1.0#	2.0#	0.013
**mRS <2 at** **6 months, n (%)**	5/10 (50.0)	17/24 (70.8)	6/12 (50.0)	0.351
**mRS (median) at** **6 months**	1.5	1.0	1.5	0.318
**mRS <2 at** **12 months, n (%)**	7/10 (70.0)	16/23 (69.6)	6/11 (54.5)	0.656
**mRS (median) at 12 months**	1.0	1.0	1.0	0.288

*adjusted standardised residual >|1.96|, equivalent to p-value < 0.05.

**adjusted standardised residual >|2.58|, equivalent to p-value < 0.01.

^#^p = 0.038 for post-hoc two-group comparison. Ab, antibodies; mRS, modified Rankin scale.

**Figure 2:**
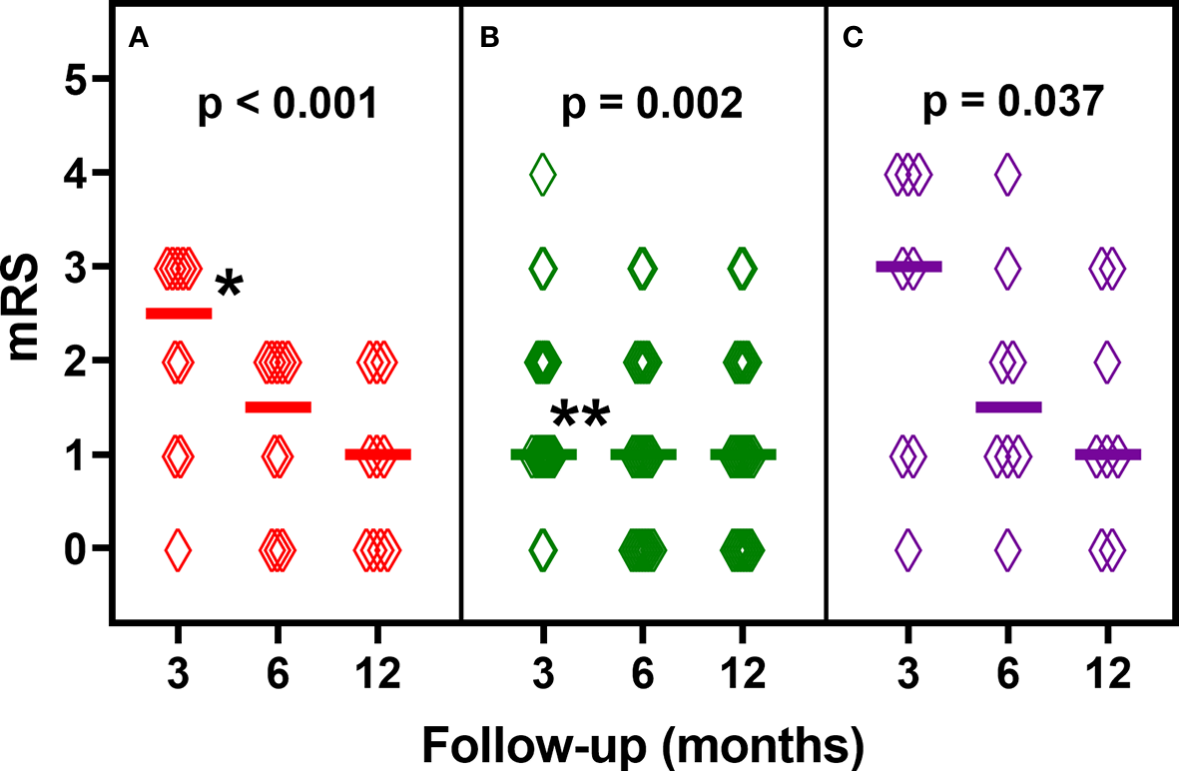
Longitudinal analysis of modified Rankin Scale (mRS) scores of patients who have completed all follow-ups at 3, 6 and 12 months: **(A)** anti-NMDAR-antibodies (n=10), **(B)** anti-LGI1/CASPR2-antibodies (n=22), **(C)** other antibodies (n=8). Diamonds indicate individual patients’ scores. Horizontal lines show median mRS scores. P-values are derived from Friedman’s test. *p < 0.0167 for post-hoc two-group comparisons (3 *vs*. 6 months and 3 *vs*. 12 months). **p < 0.0167 for post-hoc two-group comparisons (3 vs. 6 months).

**Figure 2** shows longitudinal changes of median mRS scores in these patients who have completed all follow-ups at 3, 6 and 12 months. From a median mRS of 2.5 at 3 months, patients with anti-NMDAR-Ab (n=10) improved to a median mRS of 1.5 and 1.0 at 6 and 12 months, respectively (p<0.0001). Patients with anti-LGI1/CASPR2-Ab (n=22) show a median mRS of 1 at 3, 6 and 12 months of follow-up (p=0.002). Patients with other antibodies improved from a median mRS of 3.0 at 3 months to a median mRS of 1.5 at 6 months and 1.0 at 12 months (p=0.037).

## Discussion

This prospective observational study in patients with autoimmune encephalitis covers state-of-the-art care in clinical routine in newly diagnosed patients without pre-existing disability. To our knowledge, no previous prospective studies comparing several autoimmune encephalitides are available. Our study with a structured follow-up shows several relevant findings.

First, our statistical evaluations demonstrate different dynamics in the clinical course of autoimmune encephalitides. Patients with anti-NMDAR-encephalitis show a much higher median mRS at 3 months compared to patients with anti-LGI1/CASPR2-encephalitis. The majority of anti-NMDAR-encephalitis patients received plasma exchange/immunoadsorption and second line immunosuppressive treatments at baseline. However, the high proportion of patients with significant disability at 3 months indicates the need for more efficient treatments. 70% of patients with anti-NMDAR-encephalitis in this study recover to mRS <2 at 12 months. This is in line with the reported 72.6% of cases with good outcome in a previous meta-analysis (12). A recent study in 220 anti-NMDAR-encephalitis patients reports that 86.8% of them recover to mRS <2 at 12 months follow-up (16). Patients with anti-LGI1/CASPR2-encephalitis show a much lower median mRS of 1 at 3 months. The median mRS does not change at longer follow-up time points in these patients. This may indicate that patients with anti-LGI1/CASPR2-encephalitis do not further improve at later time points. Alternatively, the mRS obtained at different time points during follow-up may be insensitive to assess neurological recovery in these patients.

Second, this prospective observational study has important implications for treatment trials. Our data indicate that clinical studies would require a sample size of about 200 patients to assess treatment outcome in patients with anti-NMDAR-encephalitis. In patients with anti-LGI1/CASPR2-encephalitis, treatment studies should focus on specific symptoms, e.g. seizures and cognition (7, 17), or use more distinctive clinical scores like the Clinical Assessment Scale in Autoimmune Encephalitis (CASE) (18).

Third, a more thorough analysis of larger cohorts of patients with anti-GABA_B_R-Ab, anti-GAD65-Ab and anti-GlyR-Ab is warranted to delineate distinctive clinical and paraclinical characteristics. In these patients, the association with cancer interferes with long-term prognosis and limits the use of immunosuppressive treatments (19).

Overall, our observations are limited to 12 months of follow-up. Longer prospective follow-up periods would be desirable. The mRS as outcome measure was used in the majority of previous studies in autoimmune encephalitis (12). We obtained this scale prospectively and did not include patients with pre-existing disability. We have captured the overall majority of patients with autoimmune encephalitis in the regions covered by the clinical centers involved in this study, but we cannot provide data about incidence and prevalence. Data about the efficacy of certain treatments cannot be derived from observations in clinical routine. In this regard, our study provides a solid basis for planning prospective treatment trials.

## Data Availability Statement

The raw data supporting the conclusions of this article will be made available by the authors, without undue reservation.

## Ethics Statement

The studies involving human participants were reviewed and approved by Ethikkommission der Medizinischen Universität Graz. The patients/participants provided their written informed consent to participate in this study.

## Author Contributions

TS-H, KE, HH, MR, HR, TB, and RH: design and concept of study, data collection and analysis, drafting and revision of manuscript. CL, SM, TM, GB, GP, RT, MG, EK, LZ, DS, AS, SO, CB, WS, FZ, JS, FD, and CE: data collection, drafting and revision of manuscript. All authors contributed to the article and approved the submitted version.

## Funding

This study was supported by a grant from the Austrian Society of Neurology (Österreichische Gesellschaft für Neurologie).

## Supplementary Material

The Supplementary Material for this article can be found online at: https://www.frontiersin.org/articles/10.3389/fimmu.2021.641106/full#supplementary-material

Supplementary Table 1 Functional outcome and immunosuppressive treatments in patients who have completed all follow-ups at 3, 6 and 12 months (Ab, antibodies; IST, immunosuppressive treatments; mRS, modified Rankin scale). Green color indicates patients with improvements in the mRS at 12 months compared to 3 months or with mRS 0 at 3, 6 and 12 months. Yellow color indicates patients with no change in the mRS at 12 months compared to 3 months. Red color indicates the patient with a worse mRS at 12 months as compared to 3 months. + immunosuppressive treatment applied, - no immunosuppressive treatment applied.

## Conflict of Interest

The authors declare that the research was conducted in the absence of any commercial or financial relationships that could be construed as a potential conflict of interest.
